# Lead remediation by geological fluorapatite combined with *Penicillium Oxalicum* and Red yeast

**DOI:** 10.1186/s12934-024-02323-2

**Published:** 2024-02-24

**Authors:** Qiang Guan, Xiaohui Cheng, Yue He, Yifan Yan, Lei Zhang, Zhan Wang, Liangliang Zhang, Da Tian

**Affiliations:** 1 Ministry of Ecology and Environment Peoples Republic of China, Nanjing Institute of Environmental Science, No. 8, Jiangwang Miao Street, Nanjing, 210042 China; 2 Anhui Province Key Lab of Farmland Ecological Conservation and Nutrient Utilization, Anhui Province Engineering and Technology Research Center of Intelligent Manufacture and Efficient Utilization of Green Phosphorus Fertilizer, College of Resources and Environment, Anhui Agricultural University, Hefei, 230036 P. R. China; 3 Key Laboratory of JiangHuai Arable Land Resources Protection and Eco-restoration, Ministry of Natural Resources, College of Resources and Environment, Anhui Agricultural University, Hefei, Anhui 230036 P. R. China

**Keywords:** EPS, Fluoroapatite, Lead remediation, Organic acids, *Penicillium oxalicum*, Red yeast

## Abstract

Phosphate solubilizing fungi *Penicillium oxalicum* (POX) and Red yeast *Rhodotorula mucilaginosa* (Rho) have been applied in Pb remediation with the combination of fluorapatite (FAp), respectively. The secretion of oxalic acid by POX and the production of extracellular polymers (EPS) by Rho dominate the Pb remediation. In this study, the potential of Pb remediation by the fungal combined system (POX and Rho) with FAp was investigated. After six days of incubation, the combination of POX and Rho showed the highest Pb remove ratio (99.7%) and the lowest TCLP-Pb concentration (2.9 mg/L). The EPS combined with POX also enhanced Pb remediation, which has a 99.3% Pb removal ratio and 5.5 mg/L TCLP-Pb concentration. Meanwhile, Rho and EPS can also stimulate POX to secrete more oxalic acid, which reached 1510.1 and 1450.6 mg/L in six days, respectively. The secreted oxalic acid can promote FAp dissolution and the formation of lead oxalate and pyromorphite. Meanwhile, the EPS produced by Rho can combine with Pb to form EPS-Pb. In the combined system of POX + Rho and POX + EPS, all of the lead oxalate, pyromorphite, and EPS-Pb were observed. Our findings suggest that the combined application of POX and Rho with FAp is an effective approach for enhancing Pb remediation.

**Supplementary Information:**

The online version contains supplementary material available at 10.1186/s12934-024-02323-2.

## Introduction

Lead (Pb) contamination is a widespread environmental issue that has garnered global attention [1, 2]. Unlike organic pollutants, Pb contamination persists in the environment without degrading over time [3]. The input pathway of Pb into the environment includes wastewater discharge, fossil fuel combustion, metal mining, and other human activities [4]. More importantly, the excessive Pb would threaten water security, causing various physical ailments such as anemia, encephalopathy, hepatitis, nephrotic syndrome, etc. [5, 6]. Therefore, the remediation of lead pollution needs to be highly valued.

Apatite has been successfully applied for heavy metal remediation in soil and water, especially for Pb contaminates [7, 8]. Fluorapatite (FAp) is the most abundant apatite in the Earth’s crust [9, 10]. The phosphorus (P) contained in FAp can react with Pb to form highly insoluble Pb minerals pyromorphite [Pb_5_(PO_4_)_3_F] [9, 10]. The formed pyromorphite (Pyro) mineral is highly stable and can lock the Pb cations in complex environments [10]. However, the low solubility of most apatites limits their application in Pb remediation [10, 11].

Phosphate solubilizing fungi (PSF) can enhance the efficiency of Pb remediation by apatite [10]. Combining PSF and apatite can significantly improve the Pb remove ratio (95%~99%) [10]. On the one hand, PSF usually has a high ability to secrete organic acids, such as oxalic acid, citric acid, etc. [12]. These organic acids can promote the release of P from apatite and react with Pb to form insoluble pyromorphite [11, 13–15]. For example, PSF-*Aspergillus niger* can secrete 2400 mg/L oxalic acid and promote 370 mg/L P release from fluorapatite, removing more than 99% of Pb cations via the form of Pyro [10]. On the other hand, the secreted oxalic acid by PSF can also react with Pb to form insoluble lead oxalate (LO) [10, 11]. Therefore, combining PSF and apatite is an efficient pathway in Pb remediation.

Like PSF, Red yeast has also been used in Pb remediation. As a widely occurring fungus, Red yeast has a strong growth rate and environmental resistance in wastewater environments [16]. However, Red yeast immobilizes Pb primarily by producing extracellular polymeric substances (EPS) [15, 17]. The produced EPS can react with Pb cations to generate EPS-Pb and reduce the toxicity of Pb [18]. In addition, adding apatite can promote EPS production by Red yeast and remove more than 98% of lead cations [19]. More importantly, EPS also contains large amounts of organic components, such as hydroxyl and carboxyl, which function in heavy metal cation chelation and supply nutrients for the growth of other microorganisms [20, 21].

*Penicillium oxalicum* (POX) and Red yeast-*Rhodotorula mucilaginosa* (Rho) are capable of surviving and maintaining their metabolite production ability under a high Pb concentration, i.e., 1000 mg/L and 2500 mg/L, respectively [11, 18]. Consequently, these two fungi have been utilized in remediating environments with high Pb levels, especially in the combination of phosphate [19, 22]. Furthermore, the high production of EPS by Rho suggests a collaborative effect between POX and Rho in Pb remediation. The combination of Rho could potentially enhance the Pb remediation by POX. However, the full potential of this combined fungal system, i.e., POX and Rho, for Pb remediation remains to be elucidated.

This study explored the Pb remediation by the combined fungal system (POX and Rho) with fluorapatite. The concentrations of P and Pb in the medium were analyzed by inductively coupled plasma optical emission spectrum (ICP-OES). Meanwhile, the toxicity characteristic leaching procedure of Pb (TCLP-Pb) in precipitates was also analyzed by ICP-OES. High-performance liquid chromatography (HPLC) was used to analyze the secretion of organic acids. The composition of precipitates was analyzed by X-ray diffraction (XRD). Rho and *P. oxalicum* morphology and mineral composition were observed using a scanning electron microscope–energy dispersive spectrometer (SEM-EDS).

## Materials and methods

### Strains incubation

*P. oxalicum* (POX) was isolated from maize rhizosphere soil in the Northern Anhui Experimental Station, Suzhou City, Anhui Province, China (Fig. S1). The collected fungi were stored in the China General Microbiological Culture Collection Center (CGMCC No. 22,475). Before the incubation, 200 g of potato was cut into small pieces and boiled with sterile water for 20 min. After filtration with four-layer sterile gauze, the filtrate was collected and mixed with 20 g agar and 20 g dextrose into a 1000 mL solution. Then, the PDA medium was separately transferred to a 250 mL conical flask and sterilized for the next experiment for 20 min at 121 ^o^C. *P. oxalicum* was cultured in potato dextrose agar medium (PDA) at 28 ^o^C for five days and then drenched with sterile water to obtain the spores. Then, mycelium fragments were filtered with four layers of sterilized cheesecloth to obtain the spore suspensions [23].

Red yeast-*Rhodotorula mucilaginosa* (Rho) (Fig. S1) was received from the China General Microbiological Culture Collection Center (CGMCC No. 16,597). Before the experiment, Rho was inoculated to a potato dextrose broth (PDB) medium (sterilized at 121^o^C for 20 min)and shaken for 48 h at 28 ^o^C, 180 rpm [15]. The preparation of the PDB medium had the same process as the PDA without agar.

### Apatite preparation

Fluorapatite (FAp) was collected from Kaiyang Phosphate Rock Reserve in Guizhou Province (N 27° 6′ 50″, E 106° 51′ 5″), China. All the FAp samples were ground to powder, dried, and filtered by 100 mesh.

### The extraction of extracellular secretions of Rho

Firstly, the incubated Rho was collected and centrifuged twice at 12,000 rpm for 20 min at 4 °C. Then, the collected Rho supernatant was added to a 3-fold volume of anhydrous ethanol. Thirdly, the mixture was allowed to rest for 48 h at 4 °C to collect extracellular secretions (EPS) and supernatant fluid (SFU). The crude extractants were transferred into 3500 molecular-weight dialysis bags and immersed in pure water for 72 h. The water was changed twice every 24 h. Finally, the extractants were moved into a 2 mL centrifuge tube for the next experiment [18].

### Pb remediation by *P. oxalicum* and Rho with FAp

The Pb contamination in water was prepared by Pb(NO_3_)_2_ powder (Xilong Scientific Ltd.). The initial Pb concentration in the medium was adjusted to 1500 mg/L. Six treatments were performed in this experiment, i.e., POX (*P. oxalicum*) + FAp, Rho + FAp, POX + Rho + FAp, POX + EPS + FAp, POX + SFU + FAp, FAp. Before the incubation, the 0.24 g Pb(NO_3_)_2_ powder and 1.0 g FAp powder were added to 250 mL Erlenmeyer flasks with 100 mL PDB medium (sterilized at 121^o^C for 20 min) in a sterile environment, respectively. Then, the 1.0 mL spore suspension (*P. oxalicum* and Rho), 1.0 mL SFU, and 1.0 g EPS were added to the corresponding treatment, respectively (Fig. S2). Each treatment was performed with four replicates. After sealing with parafilm (BS-QM-003, Biosharp), the flasks were incubated at 180 rpm, 28 ^o^C in a sterile condition. After incubation for six days, the PDB medium was filtered into 50 mL centrifugal tubes with phosphorus-free filter paper and centrifuged for 6 min at 5000 rpm. Then, the supernatant liquid was filtered through a 0.45 μm polyethersulfone (PES) membrane. The filtrates were tested for pH value, P and Pb content, and organic acids. The centrifugal precipitates were collected and dried at 55 ^o^C for 24 h to obtain the biomass, TCLP-Pb, XRD, and SEM analysis.

### TCLP-Pb extraction from precipitates

The toxicity characteristic leaching procedure (TCLP) method was used to extract the Pb from the precipitates. 2.0 g precipitates and 40mL extraction agent (select extractant 1 when pH < 5, select extractant 2 when pH > 5) were mixed and shaken at 180 rpm, for (18 ± 2) h. After centrifuge and filtering, the TCLP-Pb concentration in the precipitate was determined using ICP-OES [24]. This experiment used extraction agent 1 (pH < 5) to extract the TCLP-Pb. Extractant 1: Dissolve 5.7mL of glacial acetic acid in 500mL of deionized water, then add 1 mol/L of NaOH 64.3 ml, and bring the volume to 1 L. Adjust the pH of the solution using 1 mol/L of HNO_3_ or 1 mol/L of NaOH to maintain it within the range of 4.93 ± 0.05 [25–27].

### Instrumentation

The pH value in each treatment was measured using a pH meter (Mettler Toledo Int. Inc.). The soluble P and Pb concentrations were analyzed via ICP-OES (PerkinElmer Avio 710). The P and Pb calibration standards concentrations were 2, 5, 10, 20, 50 mg/L, and 10, 50, 100, 200, and 500 mg/L. The R square value of the external standard curve was 0.999 [28].

The contents of the organic acids were determined by high-performance liquid chromatography (HPLC) (Agilent 1200) with a column temperature of 30 °C. Then, the standard oxalic acid solution was prepared and diluted into 1000, 500, 100, 50, 20, and 10 mg/L, respectively. The R square value of the internal standard curve was 0.999 [29].

The mineralogical characterization of the precipitates was examined by Rigaku D/Max-2500 X-ray diffraction (Cu-Kα; 36 kV; 20 mA; scanned from 5° to 60° at a speed of 4° s^− 1^). The XRD patterns were analyzed by MDI Jade 6.5 software for phase identification.

The morphology of Rho and minerals was observed by SEM (S4800 Hitachi) with an acceleration voltage of 3 kV. The samples were coated with a layer of gold for 1 min in Hitachi E-1010 Sputter to enhance image quality.

## Results

### pH and dry biomass in medium

**Fig. 1:**
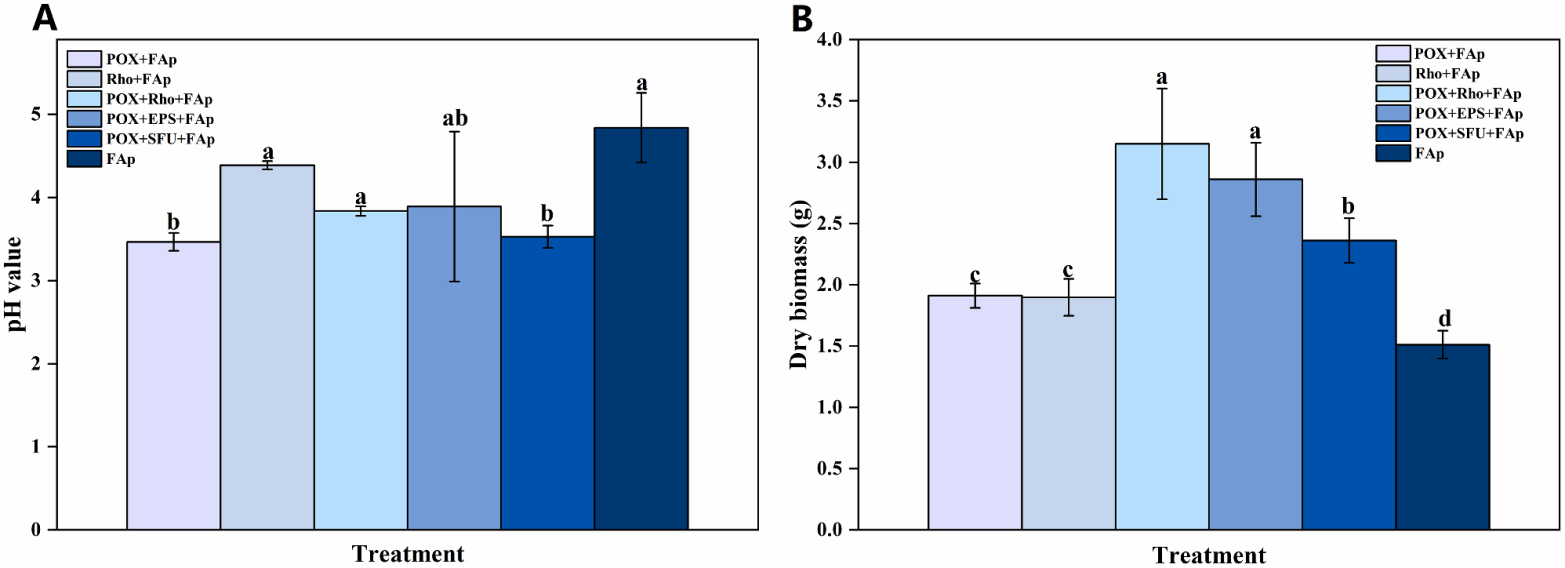
The pH value (**A**) and dry biomass (**B**) in each treatment after six days of incubation. The error bars represent the standard deviations of four replicates. The significant differences among the treatments were identified by Tukey’s honestly significant difference test (*p* < 0.05) via one-way ANOVA. Rho: *Rhodotorula mucilaginosa*; POX: *P. oxlaicum*

The initial pH in the medium was 5.7. After six days of incubation, the pH value in all treatments was decreased to 3.5–3.8 (Fig. 1A). In the POX + FAp and Rho + FAp treatments, the pH dropped to 3.5 and 4.4 (Fig. 1A). In POX + Rho + FAp, POX + EPS + FAp, and POX + SFU + FAp, the pH value was 3.8, 3.9, and 3.5 after six days of incubation, respectively (Fig. 1A). The pH in FAp treatment also decreased to 4.8 (Fig. 1A).

In POX + FAp, Rho + FAp, and FAp treatments, the dry biomass was 1.9 g, 1.8 g, and 1.2 g after six days of incubation, respectively (Fig. 1B). In POX + EPS + FAp and POX + SFU + FAp treatments, the dry biomass after six days of incubation was 2.9 g, and 2.4 g, respectively (Fig. 1B). The POX + Rho + FAp treatment had the highest dry biomass compared with other treatments, reached to 3.1 g after six days of incubation (Fig. 1B).

### P concentration and oxalic acid concentration

**Fig. 2:**
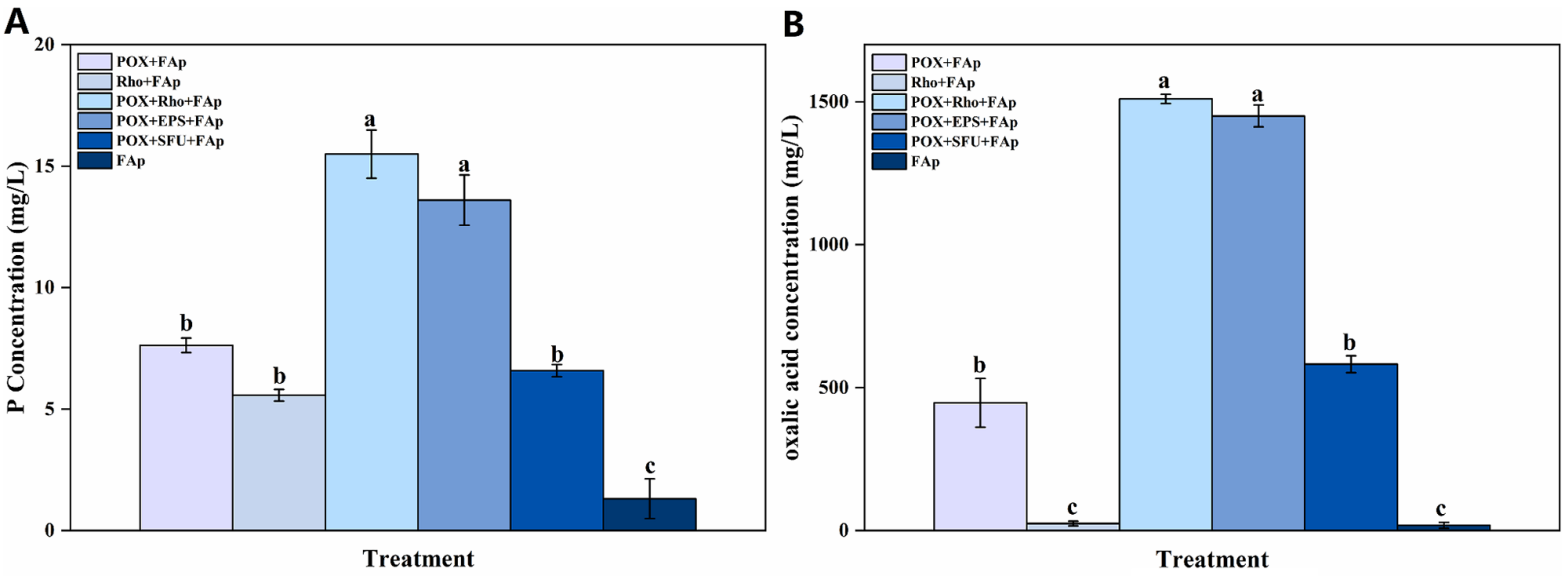
The P concentration (**A**) and oxalic acid (**B**) concentration in each treatment after six days of incubation. The error bars represent the standard deviations of four replicates. The significant differences among the treatments were identified by Tukey’s honestly significant difference test (*p* < 0.05) via one-way ANOVA. Rho: *Rhodotorula mucilaginosa*; POX: *P. oxlaicum*

After six days of incubation, POX + Rho + FAp and POX + EPS + FAp treatments had a high P concentration, i.e., 16.1 and 14.2 mg/L, respectively (Fig. 2A). In POX + FAp, Rho + FAp, and POX + SFU + FAp treatments, the P concentration had no significant difference, i.e., 8.3, 6.1, and 6.9 mg/L, respectively (Fig. 2A). In FAp treatment, the P concentration had the lowest value of 1.3 mg/L (Fig. 2A).

Oxalic acid is the primary organic acid in each treatment. In POX + Rho + FAp and POX + EPS + FAp treatments, the oxalic acid concentration had the highest value of 1510.1 and 1450.6 mg/L at six days, respectively (Fig. 2B). The oxalic acid concentration in POX + FAp and POX + SFU + FAp treatments was 446.6 and 581.8 mg/L after six days of incubation (Fig. 2B). In Rho + FAp and FAp treatment, the oxalic acid almost undetectable after six days of incubation, i.e., 24.6 and 18.4 mg/L (Fig. 2B).

### Pb, TCLP-Pb concentration, and pb remove ratio

**Fig. 3:**
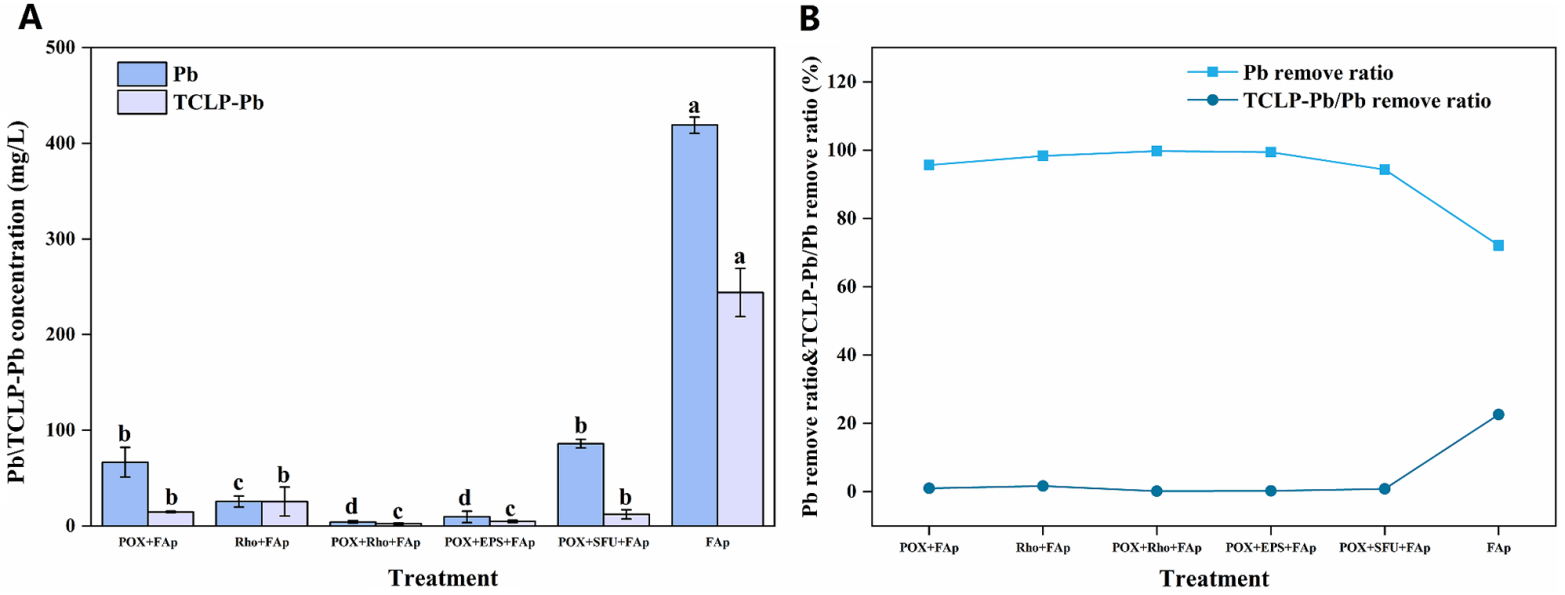
The Pb and TCLP-Pb concentration (**A**) and Pb remove ratio (**B**) in each treatment after six days of incubation. The error bars represent the standard deviations of four replicates. The significant differences among the treatments were identified by Tukey’s honestly significant difference test (*p* < 0.05) via one-way ANOVA. Rho: *Rhodotorula mucilaginosa*; POX: *P. oxlaicum*

The initial Pb concentration in the PDB medium was 1500 mg/L. After six days of incubation, the Pb concentration in POX + Rho + FAp and POX + EPS + FAp treatments decreased to the lowest value of 4.7 and 9.8 mg/L (Fig. 3A). In POX + FAp, Rho + FAp, and POX + SFU + FAp treatments, the Pb concentration decreased to 78.5, 27.4 and 88.5 mg/L, respectively (Fig. 3A). However, the Pb concentration in FAp treatment had the highest value of 430.1 mg/L after six days of incubation (Fig. 3A). The TCLP-Pb concentration from precipitates in each treatment was significantly lower than Pb concentration in the medium. After six days of incubation, the TCLP-Pb concentration in POX + Rho + FAp and POX + EPS + FAp treatments was only 2.9 and 5.5 mg/L (Fig. 3A). In POX + FAp, Rho + FAp, and POX + SFU + FAp treatments, the TCLP-Pb concentration was 15.7, 30.1, and 12.9 mg/L, respectively (Fig. 3A). Similarly, the TCLP-Pb in FAp treatment also had the highest value of 257.3 mg/L (Fig. 3A).

The Pb removal ratio in POX + FAp, Rho + FAp, POX + Rho + FAp, POX + EPS + FAp, and POX + SFU + FAp treatments had a close value of 95.6%, 98.2%, 99.7%, 99.3%, and 94.1%, respectively (Fig. 3B). In FAp treatment, the Pb remove ratio had the highest value of 71.3% after six days of incubation (Fig. 3B). That is to say, more than ~ 1000 mg/L Pb was removed from the medium to the precipitates in each treatment. However, the stable capacity of removed Pb in each treatment was different. The TCLP-Pb/removed Pb ratio had the lowest value in POX + Rho + FAp and POX + EPS + FAp treatments, i.e., 0.19% and 0.37% (Fig. 3B). In POX + FAp, Rho + FAp, and POX + SFU + FAp treatments, the TCLP-Pb/removed Pb ratio was 1.10%, 2.04%, and 0.91%, respectively (Fig. 3B). In FAp treatment, the TCLP-Pb/removed Pb ratio had the highest value of 24.05% (Fig. 3B).

### XRD analysis

**Fig. 4:**
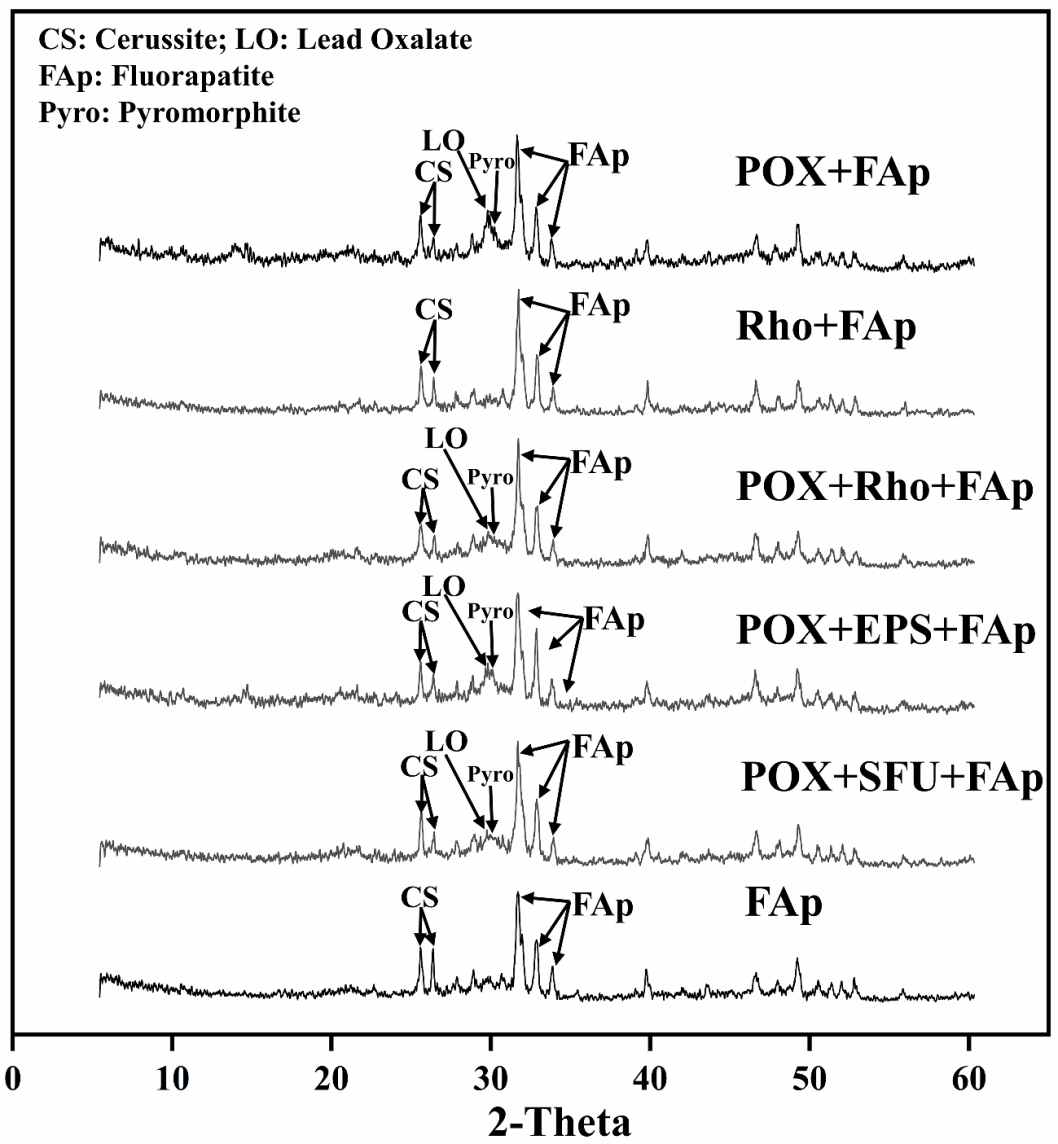
XRD patterns of precipitation in each treatment after six days of incubation

Figure 4 shows the XRD patterns of precipitates in each treatment after six days of incubation. The peak of FAp (31.94°, 32.27°, and 33.12°) and Cerussite (24.8° and 25.49°) were observed in each treatment (Fig. 4). The peak located at 29.68° stand for lead oxalate mineral was detected in POX + FAp, POX + Rho + FAp, POX + EPS + FAp, and POX + SFU + FAp treatments (Fig. 4). In addition, the peaks of pyromorphite (Pyro) (30.74° and 30.84°) also appeared in POX + FAp, POX + Rho + FAp, POX + EPS + FAp, and POX + SFU + FAp treatments (Fig. 4).

### Full width at half maxima (FWHM) value and peak area ratio

**Fig. 5:**
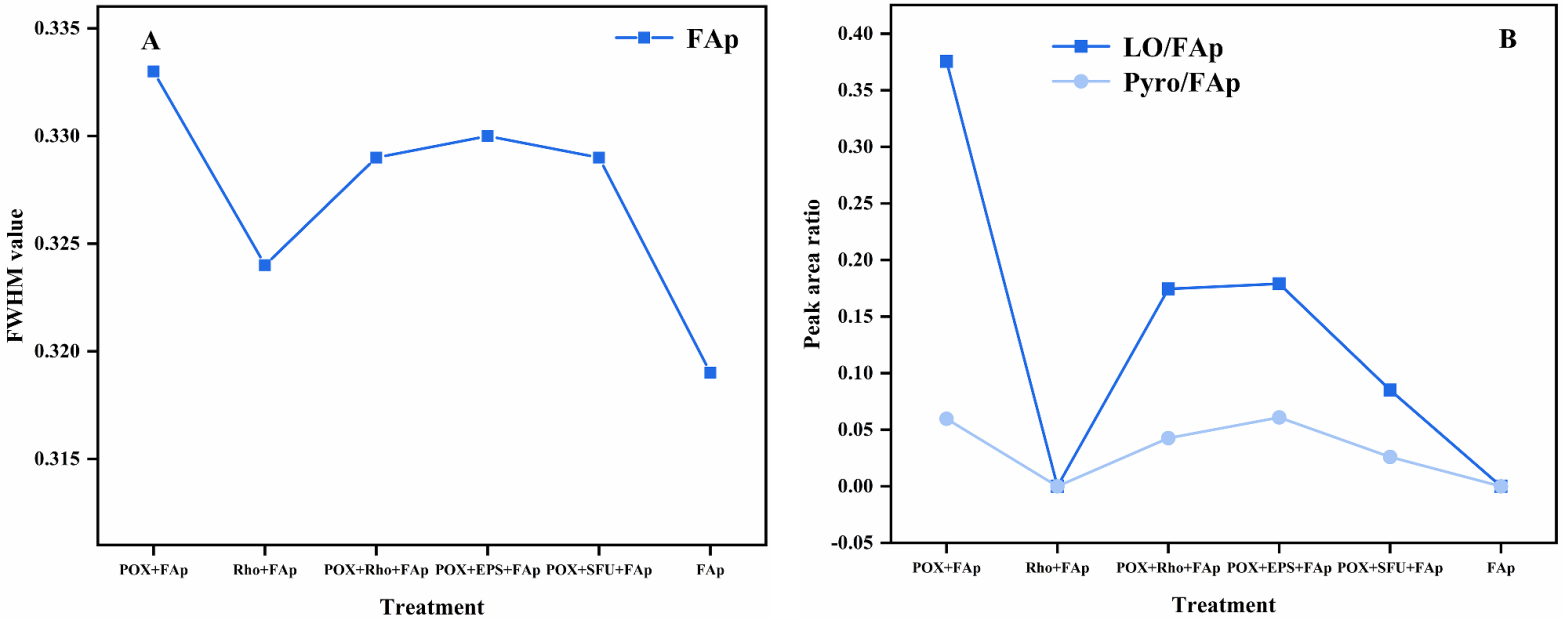
The FWHM value of FAp (**A**) and peak value ratio of LO/FAp and Pyro/FAp (**B**) in each treatment after six days of incubation. FAp: fluorapatite, LO: lead oxalate, Pyro: pyromorphite

The FWHM value in POX + FAp, POX + Rho + FAp, POX + EPS + FAp, POX + SFU + FAp, and FAp treatments was 0.333, 0.324, 0.329, 0.330, 0.329 and 0.319 after six days of incubation, respectively (Fig. 5A). The peak area ratio of LO/FAp in POX + FAp, POX + Rho + FAp, POX + EPS + FAp, and POX + SFU + FAp treatments was 0.376, 0.175, 0.179, and 0.085 after six days of incubation, respectively (Fig. 5B). The peak area ratio of Pyro/FAp in POX + FAp had the highest value of 0.060 (Fig. 5B). In POX + Rho + FAp, POX + EPS + FAp, and POX + SFU + FAp treatments, the Pyro/FAp peak area ratio was 0.043, 0.061, and 0.025 after six days of incubation, respectively (Fig. 5B).

### SEM-EDS analysis

**Fig. 6:**
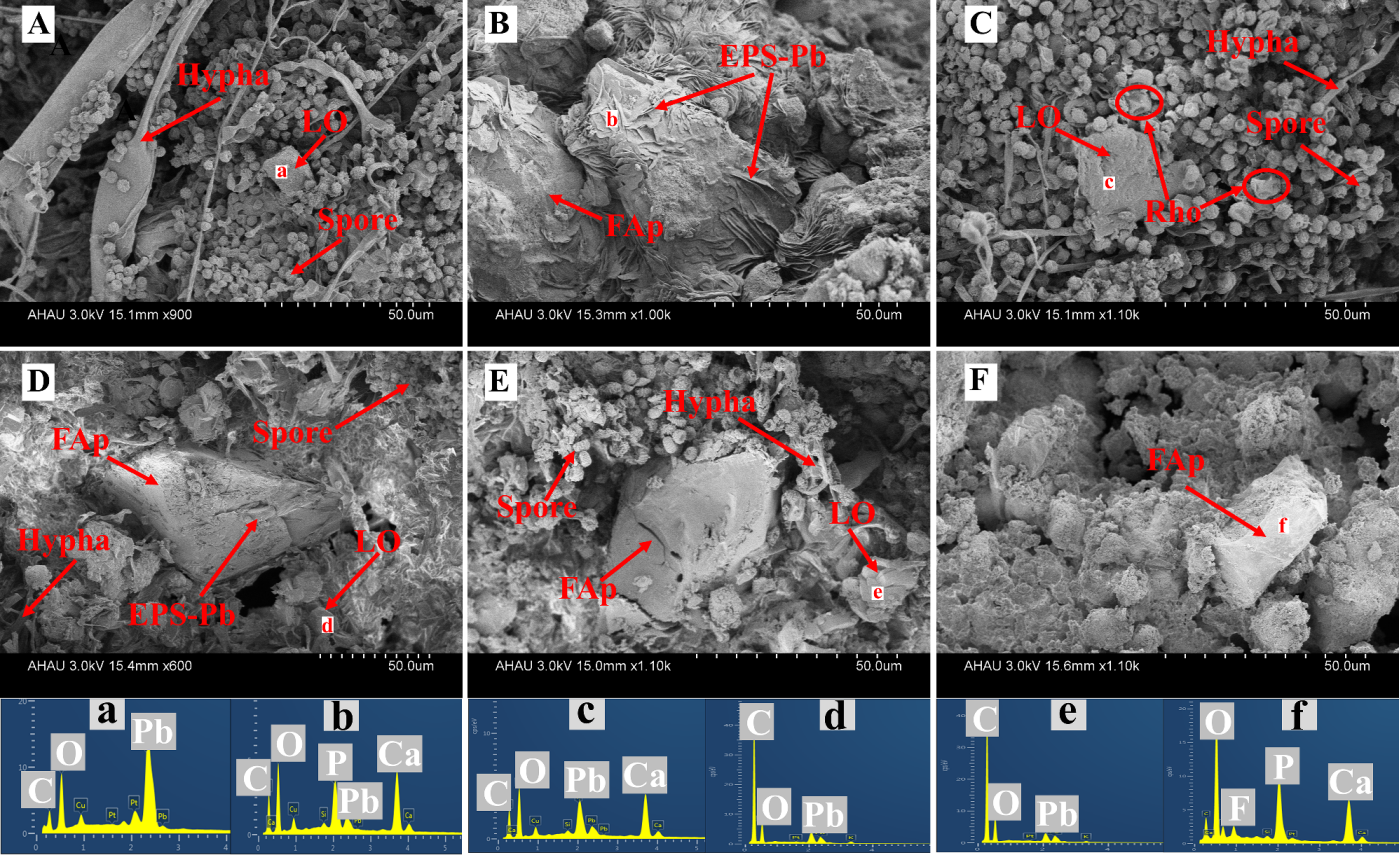
SEM images and EDS point data in POX + FAp (**A**), Rho + FAp (**B**), POX + Rho + FAp (**C**), POX + EPS + FAp (**D**), POX + SFU + FAp (**E**), FAP (**F**) treatments after six days of incubation. The symbols (**a**,**b**,**c**,**d**,**e**,**f**) stand for the point area of EDS analysis in each treatment. LO: lead oxalate, FAp: fluorapatite, Rho: *Rhodotorula mucilaginosa*

The SEM-EDS images of fungi and mineral morphologies are shown in Fig. 6. The spores and hypha of *P. oxalicum* can be observed in the treatment with adding POX (Fig. 6). Meanwhile, the Rho was also observed in POX + Rho + FAp treatment and had a significant size than *P. oxalicum* spores (Fig. 6C). In each treatment, the mineral of FAp can be observed with a large size (Fig. 6). The mineral of LO can also be observed in POX + Rho + FAp, POX + EPS + FAp, and POX + SFU + FAp treatments (Fig. 6). Large amounts of EPS-Pb was observed in Rho + FAP treatment (Fig. 6B). In POX + EPS + FAp treatment, both of LO and EPS-Pb were observed (Fig. 6D).

## Discussion

Apatite is an efficient material in Pb remediation due to its ability to reduce the bioavailability of Pb through mineralization and adsorption [30]. For example, using crystallite-size apatite (200 × 40 nm) can immobilize Pb by forming a pyromorphite mineral [31]. Meanwhile, the apatite can also adsorb ~ 0.16 mmol Pb/g in an aqueous solution [32]. However, the mineral pyromorphite is challenging to form due to the low solubility of FAp [33]. Our results indicate that only 71.3% of Pb cations (1069 mg/L) were removed by only FAp, with more than 430 mg/L Pb cations also remaining in the solution (Fig. 3). Additionally, these removed Pb cations were unstable and could be released again in an acidic environment. The TCLP-Pb results indicated that ~ 24.1% Pb (257.3 mg/L) cations are released from the removed Pb (1069 mg/L) (Fig. 3). Therefore, using FAp in Pb remediation needs further reinforcement, especially in phosphorus release and Pb minerals formation.

The application of fungi of *P. oxalicum* and Rho can promote Pb remediation by FAp [14, 15, 34]. Compared to using FAp alone, the combination of *P. oxalicum* and Rho increased the Pb remove ratio to 95.6% and 98.2%, respectively (Fig. 3). This Pb remove ratio is similar to the previous research, i.e., ~ 98% Pb remove ratio by *P. oxalicum* and Rho with phosphate [19, 22]. Meanwhile, *P. oxalicum* and Rho can survive under the 1500 mg/L Pb concentration [19, 22]. The hypha and spores can be observed in SEM images in each treatment (Fig. 6). In addition, the Pb cations immobilization in *P. oxalicum* and FAp combination system is *via* the formation of pyromorphite and lead oxalate, while in Rho and FAp is primarily through the formation of EPS-Pb. The XRD and SEM-EDS results can also indicate the production form of removed Pb cations (Figs. 4 and 6). Therefore, the results suggest that the Pb remediation pathways between *P. oxalicum* and Rho with FAp are different.

The different secondary metabolites between *P. oxalicum* and Rho decide the final form of removed Pb cations. Like other PSFs, *P. oxalicum* can secrete large amounts of organic acid, especially oxalic acid, which is the primary organic acid secreted by these fungi [23, 35]. Oxalic acid not only promotes the release of P in FAp to form pyromorphite (Pyro) but also can directly combine Pb to form insoluble lead oxalate (LO) [10, 36–38]. The XRD results also indicate the formation of LO and Pyro (Fig. 4). Meanwhile, both LO and Pyro are stable and have a low K*sp* value (~ 10^− 11^ and ~ 10^− 85^) [10, 22]. Hence, only 1.1% of Pb cations (TCLP-Pb) can be released from removed Pb in POX + FAp treatment (Fig. 3B). Unlike *P. oxalicum*, Rho with FAp in Pb immobilization is primary through the production of EPS rather than the dissolution of FAp [19]. In Rho + FAp treatment, the FWHM value of FAp is lower than POX + FAp treatment, which could indicate the weakly dissolution of FAp. The SEM images also confirmed the production of EPS and the formation of large amounts of EPS-Pb (Fig. 6B). EPS has strong binding and adsorption ability for metal cations due to its complex functional groups [39]. More importantly, adding phosphate can increase Rho’s production of EPS [19]. Meanwhile, the EPS is more efficient in binding with heavy metal cations (e.g., Cu^2+^ and Pb^2+^) [40, 41]. Therefore, Rho is more efficient than *P. oxalicum* in Pb remediation. Our results also confirmed that the Pb remove ratio in Rho + FAp is higher than POX + FAp, i.e., 98.2% vs. 95.6% (Fig. 3B).

Compared with *P. oxalicum* and Rho, the combined fungal system (*P. oxalicum* + Rho) is more efficient in Pb remediation by FAp. Our results indicate that POX + Rho + FAp has the highest Pb remove ratio (99.7%) and the lowest TCLP-Pb concentration (Fig. 3). Rho can promote Pb remediation by *P. oxalicum* through the secretion of EPS. It has a similar Pb remove ratio (99.7% and 99.3%) and TCLP-Pb concentration (2.9 and 5.5 mg/L) between POX + Rho and POX + EPS treatment (Fig. 3). On the one hand, EPS has an complex mixture of multiple biomolecules (proteins, polysaccharides, humic-like substances, uronic acid, nucleic acid, lipids and glycoproteins) [15, 42, 43]. These biomolecules can promote the secretion of oxalic acid by *P. oxalicum* (Fig. 2B). The secreted oxalic acid can react with Pb to form lead oxalate and promote FAp transfer to pyromorphite [22]. The analysis of LO/FAp and Pyro/FAp peak area ratio in POX + Rho + FAp and POX + EPS + FAp are higher than in POX + SFU + FAp treatment (no EPS) (Fig. 5). Therefore, Rho combined with PSF that have higher oxalic acid secretion ability (e.g., *Aspergillus niger*) could be more effective in Pb remediation by FAp [23, 35]. On the other hand, the EPS has a complex molecular composition, so it has strong adsorption and combining capacity to lead cations and can react with Pb to form a stable EPS-Pb [18, 44, 45]. The formed EPS-Pb reduced the Pb toxicity and promoted the growth of *P. oxalicum* (Fig. 1B). Studies have pointed out that proteins and polysaccharides in EPS are the main factors of resistance to heavy metals [42, 46]. These organic components have highly branched chemical structures and functional groups, such as hydroxyl and carboxyl [44, 45]. This spatial structure and complex composition allow it to adsorb and chelate with Pb^2+^, thereby reducing the toxicity of heavy metals [15].

## Conclusion

PSF *P. oxalicum* and Red yeast Rho combined with FAp can significantly promote the Pb remediation and maintain the stability of removed Pb cations. Oxalic acid secreted by *P. oxalicum* not only promotes P release from FAp to form pyromorphite but also reacts with Pb to form insoluble lead oxalate. EPS dominates the Pb remediation by Rho via the formation of stable EPS-Pb. More importantly, the secreted EPS by Rho can promote the growth of *P. oxalicum* and increase the secretion of oxalic acid. The combination of *P. oxalicum* and Rho is the most effective pathway in Pb remediation by FAp.

### Electronic supplementary material

Below is the link to the electronic supplementary material.

**Supplementary Material 1: Additional file 1: Fig. S1.** Phosphate solubilizing fungi *Penicillium oxalicum* and Red yeast *Rhodotorula mucilaginosa* were used in this experiment. **Fig. S2.** The sketch of each treatment in the experiment. **Fig. S3.** The flask experiment images after six days of incubation.

## Data Availability

The publication includes a list of all the datasets used in this investigation.

## Abbreviations

POX: *Penicillium oxalicum*
Rho: *Rhodotorula mucilaginosa*
EPS: extracellular polymers
FAp: fluorapatite
TCLP-Pb: toxicity characteristic leaching procedure of Pb
PSF: phosphate solubilizing fungi
LO: lead oxalate
SFU: supernatant fluid
PDB: potato dextrose broth
Pyro: pyromorphite
