# Supplementary material for: Lead remediation by geological fluorapatite combined with Penicillium Oxalicum and Red yeast
Source: Microb Cell Fact. 2024 Feb 24;23:64. doi: 10.1186/s12934-024-02323-2 (PMC10893623; doi:10.1186/s12934-024-02323-2)
Supplement: Supplementary file 1 — Supplementary Material 1: Additional file 1: Fig. S1. Phosphate solubilizing fungi Penicillium oxalicum and Red yeast Rhodotorula mucilaginosa were used in this experiment. Fig. S2. The sketch of each treatment in the experiment. Fig. S3. The flask experiment images after six days of incubation. [file 12934_2024_2323_MOESM1_ESM.docx]

**Lead remediation by** **geological fluorapatite combined with *Penicillium oxalicum* and Red yeast**

Qiang Guan ^1#^, Xiaohui Cheng^2,3#^, Yue He^1^, Yifan Yan^1^, Lei Zhang^1*^, Zhan Wang^1^, Liangliang Zhang^2,3^, Da Tian^2,3*^

^1^ Ministry of Ecology and Environment Peoples Republic of China, Nanjing Institute of Environmental Science, No. 8, Jiangwang Miao Street, Nanjing 210042, China

^2^ Anhui Province Key Lab of Farmland Ecological Conservation and Nutrient Utilization, Anhui Province Engineering and Technology Research Center of Intelligent Manufacture and Efficient Utilization of Green Phosphorus Fertilizer, College of Resources and Environment, Anhui Agricultural University, Hefei, 230036, P. R. China.

^3^ Key Laboratory of JiangHuai Arable Land Resources Protection and Eco-restoration, Ministry of Natural Resources, College of Resources and Environment, Anhui Agricultural University, Hefei, 230036, P. R. China.

**Corresponding to:**

**Lei Zhang**

Ministry of Ecology and Environment Peoples Republic of China, Nanjing Institute of Environmental Science, Nanjing, Jiangsu, 210042, China. E-mail: lizaoyutian@126.com

**Da Tian**

College of Resources and Environment, Anhui Agricultural University, Hefei, Anhui, 230036, China. E-mail: tianda@ahau.edu.cn

^#^ These authors have contributed equally to this work and share the first authorship


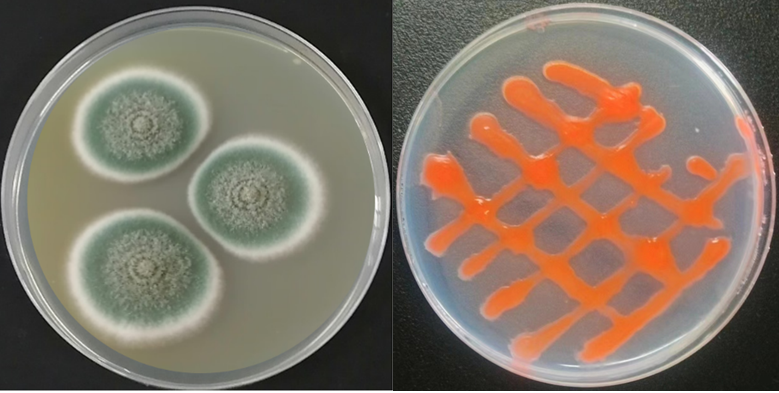


Fig. S1. Phosphate solubilizing fungi *Penicillium oxalicum* and Red yeast *Rhodotorula mucilaginosa* were used in this experiment.


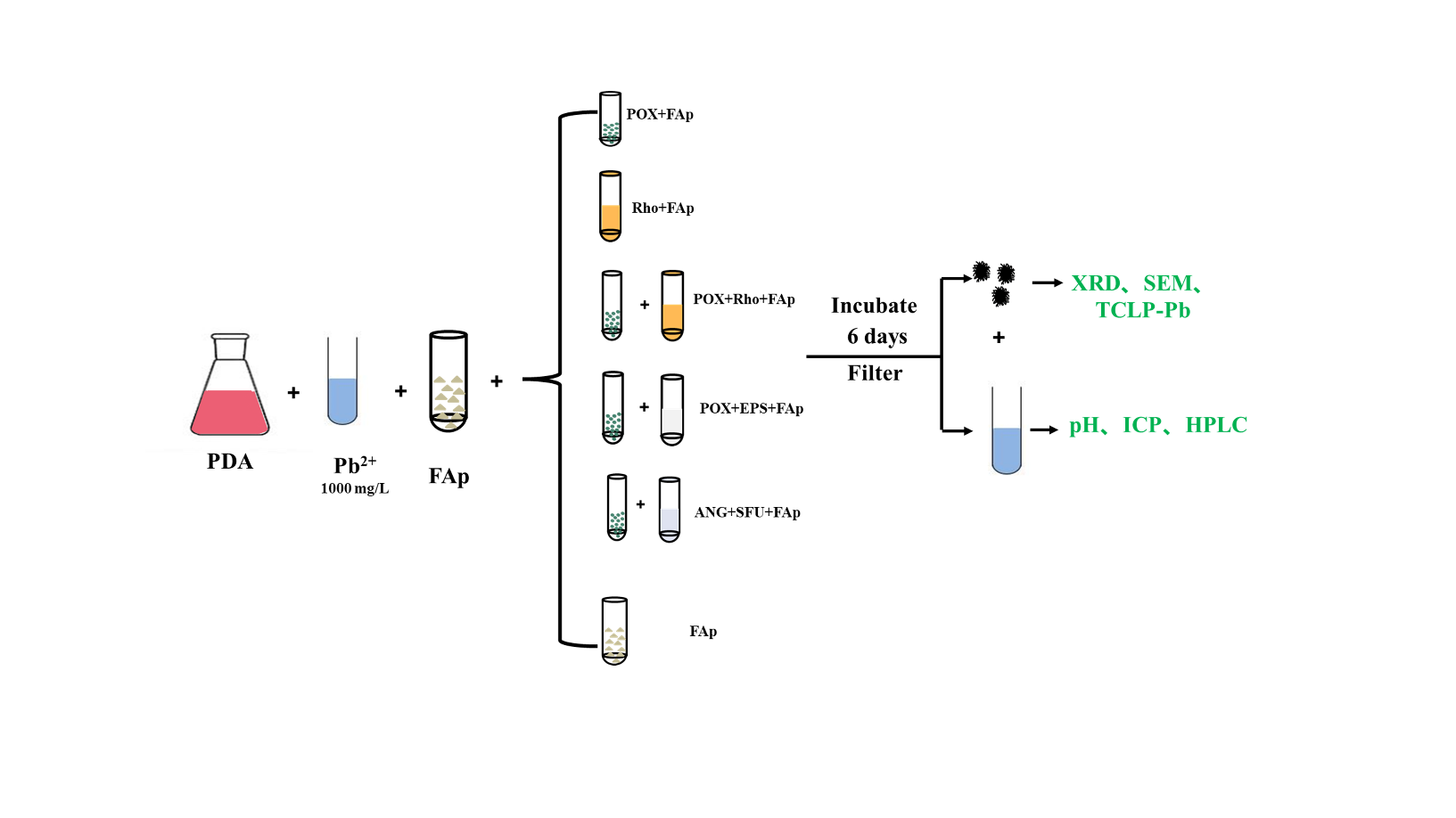


Fig. S2. The sketch of each treatment in the experiment.


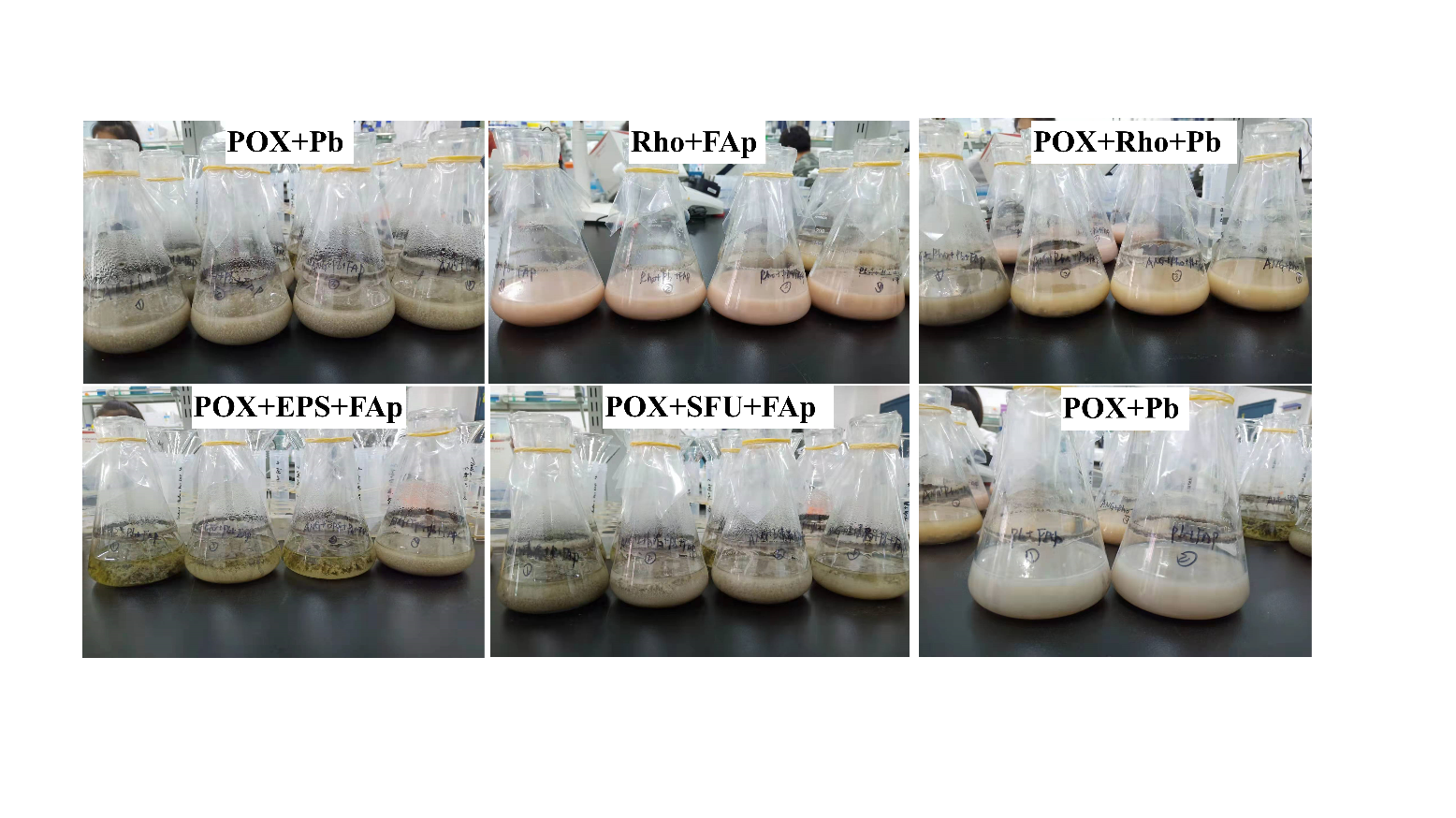


Fig. S3. The flask experiment images after six days of incubation.
